# Analysis of the gender-specific risk factors of social anxiety among left-behind middle school students in Deyang

**DOI:** 10.3389/fpsyt.2026.1780497

**Published:** 2026-03-25

**Authors:** Feng Wu, Wei Qiu, Xue Xie, Qin Qin, Mei Yi, Li Zeng, Qiang Huang

**Affiliations:** 1 Department of Psychiatry, The Psychiatric Hospital of Zhongjiang, Deyang, China; 2 Education and Sports Bureau of Zhongjiang, Deyang, China

**Keywords:** communication method, extracurricular activities, gender, left-behind middle school students, social anxiety

## Abstract

**Background:**

Left-behind children in China often experience mental health challenges, including social anxiety (SA). Gender-related factors may play a key role in the development of these issues. This study aims to investigate gender-specific risk factors associated with SA among left-behind middle school students (LBMSSs) in Deyang. The findings of this study will contribute to the development of targeted intervention strategies tailor to the unique needs of this population.

**Method:**

From March to May of 2025, a questionnaire survey was conducted among LBMSSs in grades 7–9 from 20 middle schools by a stratified cluster sampling method. A total of 780 LBMSSs (431 males and 349 females) were included in the final analysis. The Chinese version of the Children’s Social Anxiety Scale was used to assess SA levels, and binary logistic regression was employed to examine gender-specific risk factors.

**Results:**

A total of 331 individuals exhibited SA symptoms, with a detection rate of 42.4%. A substantial interaction between gender and SA was identified (OR = 0.615, *P* = 0.001). Among female students, extracurricular activities (*OR* = 0.301, P = 0.005), paternal contacting frequency (*OR* = 0.817, *P* = 0.035), and video communication (*OR* = 0.423, *P* = 0.021) emerged as protective factors. Conversely, neglecting parenting (*OR* = 2.170, *P* = 0.032) was identified as a risk factor. Among male students, the occurrence of significant events in the past six months (*OR* = 2.662, *P* = 0.001) was a risk factor. In contrast, democratic parent-child communication (*OR* = 0.538, *P* = 0.037) was a protective factor.

**Conclusion:**

A notable distinction emerges in the underlying factors contributing to SA among LBMSSs, with marked gender disparities. The development of specialized psychological intervention strategies tailored to the distinct needs of both genders is imperative. For girls, promoting extracurricular activities, enhancing father involvement, and mitigating neglect are crucial. Conversely, for boys, the provision of psychological support following significant events and the cultivation of democratic parent-child communication methods should be prioritized.

## Introduction

In China, the ongoing process of urbanization has led to the migration of surplus rural laborers to cities, thereby forming a distinctive social group known as “left-behind children (LBC).” LBC are minors under the age of 16 whose parents, either both or one, are employed away from the child’s residence, thereby preventing the other parent from providing guardianship (1). According to the 2025 China Statistical Yearbook (2), 3.74 million LBC enrolled in junior high school nationwide in 2024, accounting for 7% of the total, including 1.73 million females. Despite declines in the overall number and proportion of LBC in school enrolment in recent years (**Supplementary Figures 1A, B**), the problem of LBC remains salient in economically underdeveloped areas of western China (3), particularly in rural regions. The population of this demographic in western rural areas is increasing gradually, thereby exerting considerable pressure on local social and economic development.

LBC are more prone to being neglected for long-term separation from their parents (1), which in turn can induce various negative psychological states, manifested as a significant increase in the risk of depression, anxiety, self-injurious behaviors, or loneliness (2, 3). Among these, social anxiety (SA)—characterized by fear of negative evaluation and social avoidance—is a particularly salient concern, which is an overlooked psychological issue in children and adolescents. 6-15-year-old Chinese children revealed a SA prevalence of 23% (95%CI=15.4%-26.9%) (4), with an increase in prevalence observed following the emergence of epidemics associated with the novel coronavirus (SARS-CoV-2) (5, 6). Notably, 35% of the LBC may exhibit SA (7). Furthermore, a discrepancy was observed in the methods employed by girls and boys in response to adverse emotions (8), which was found to be inconsistent with the development of mental health across individuals (9). Therefore, we propose that the protective or risk factor of developing SA may be affected by gender in left-behind middle school students (LBMSSs). In the following study, we chose the LBMSSs in the Deyang region and delved into the influencing factors of anxiety emotions across different genders, to provide scientific evidence for the development of evidence-based intervention strategies tailored to the local context.

## Methods

### Participants

The present study employs a cross-sectional research design to investigate the mental health of LBMSSs (grades 7-9) during March to May of 2025. Stratified cluster sampling was employed, with the 47 middle schools in Zhongjiang serving as the sampling frame. The survey of 20 schools was to be conducted across the 20 regional divisions, with one school from each division. These schools were divided into three strata based on the size of the enrolled student population (27 schools were <500, 13 schools were 500-1000, and 7 schools were >1000), with the selection of 15, 4, and 1 school in each stratum, respectively. According to the number of LBMSSs and total population of students provided by the Deyang Education Bureau, under the tolerance error (δ) of 2%, the significance level of 0.05, and considering 10% loss. Subsequently, a total of 951 LBMSSs were estimated to be surveyed (with 513, 387, and 51 in each stratum, respectively). Children who had been left behind were randomly selected from each school in each tier in equal proportion. Finally, within each selected school, a corresponding number of students were randomly selected from the list of LBMSSs with equal probability to complete the assessment. Informed consent was obtained from the research subjects or their guardians, and this project was approved by the Ethics Committee of Zhongjiang County Psychiatric Hospital (No. llsc-llej-20241220(1225)-01B). The design, implementation, and reporting of this study are in strict accordance with the ethical guidelines of the Declaration of Helsinki and its subsequent amendments.

Inclusions: 1) LBC refers to children separated from one or both parents due to parental migration for employment. This migration, whether temporary or long-term, results in the children remaining in their registered residence, unable to live with both parents. 2) Grades 7–9 are to be considered. 3) Individuals with non-local registered residence who have resided in Zhongjiang for a cumulative period of six months or more within the past 12 months are to be considered. Alternatively, those with locally registered residences who have been away from the county for less than six months are also eligible.

Exclusions: 1) Those who have been separated from their township (community) for a period exceeding half a year. 2) Those who have dropped out of school for various reasons, irrespective of their household registration status within their respective township (community).

### Data collection

The participants in the survey comprise all personnel who have completed course training and passed the relevant assessments. Data are collected via electronic questionnaires, which respondents complete independently under the guidance of the surveyors. During the survey, the surveyors answer questions and address any questions. After the questionnaires are completed, they are promptly verified in a unified manner.

Collect demographic information: gender, age (years), ethnicity; family structure characteristics: only child, number of family members living together, etc.; parents’ absence status: type of parents’ absence (both parents/father only/mother only), duration of absence (years), frequency of contact, etc.

The SA level of LBC was assessed using the Chinese version of the Social Anxiety Scale for Children (SASC) (10). It encompasses two dimensions: fear of negative evaluation and social avoidance and distress, with a total of 10 items and two factors: fear of negative evaluation (items 1, 2, 5, 6, 8, 10) and social avoidance and distress (items 3, 4, 7, 9). A higher score indicates a more severe level of anxiety, with a score of 8 or above defined as high social anxiety. The Cronbach’s alpha coefficient of this scale is 0.884, indicating good reliability.

### Statistics

The statistical analysis was conducted using SPSS 26 (IBM Corp., NY). Categorical variables were analyzed using the chi-square test or the Mann-Whitney test. Continuous variables were tested using the independent sample *t*-test if they met normality and homogeneity of variance; otherwise, the Mann-Whitney test was used. Spearman’s test is used to analyze the correlation between SA and other variables. Binary logistic regression was used for risk factor analysis, and the Cox & Snell *R*^2^ was used to evaluate the model’s goodness of fit. P<0.05 was considered statistically significant.

## Results

**Figure 1:**
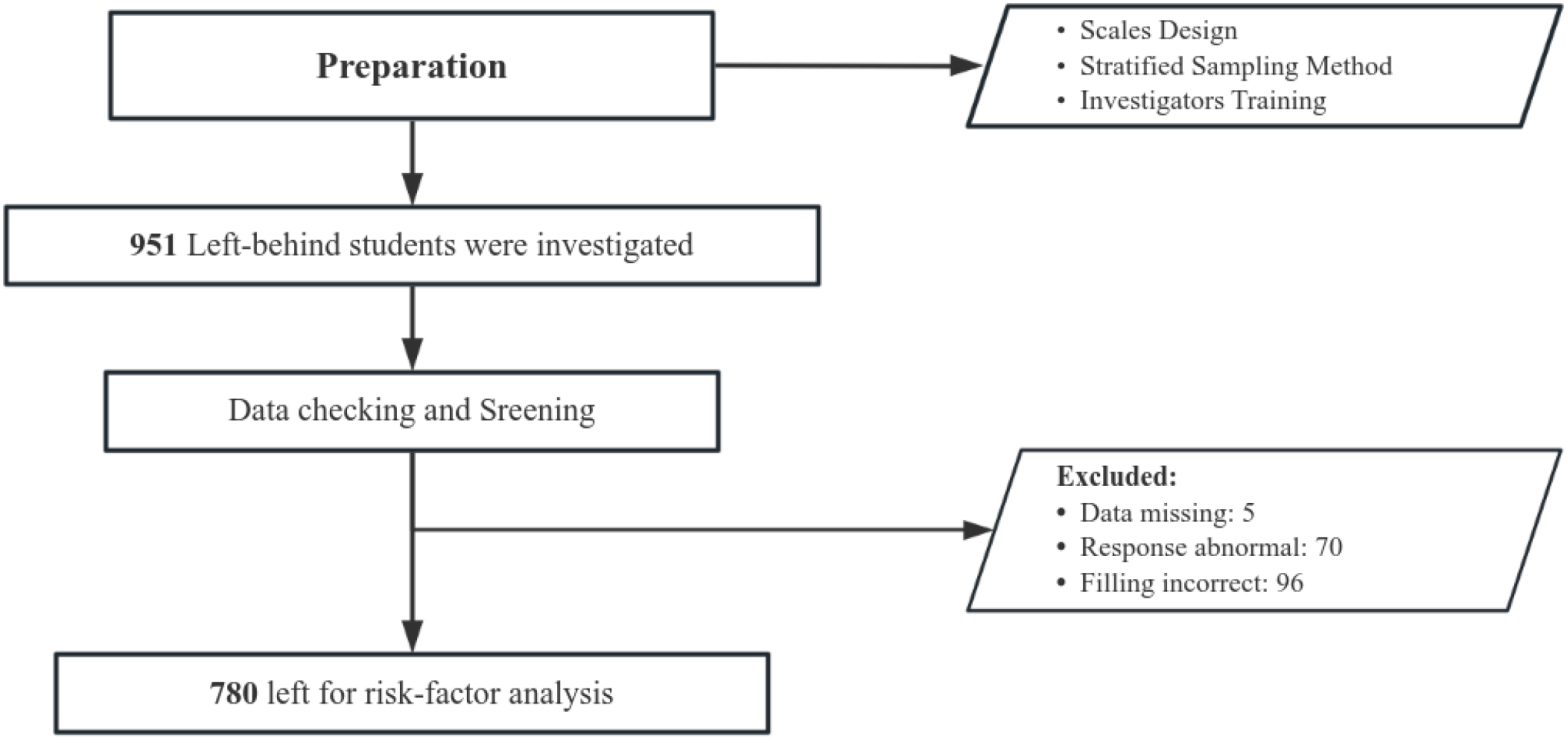
The data collection and screening process for the left-behind middle school students investigated in this study.

A total of 951 individuals were surveyed, and 946 questionnaires were successfully collected. Following exclusion of 70 participants with abnormal responses (average response time < 1s per item) and 90 with incomplete data, the final analysis included 780 participants: 331 in the SA group and 449 in the normal group. The sample included 431 males and 349 females. A statistically significant interaction was identified between gender and social anxiety (*OR*_Gender×SA_=0.615, *P* = 0.001). The participant survey screening process is shown in **Figure 1**.

Among female LBMSSs, there were statistically significant differences in anxiety subgroups regarding participation in extracurricular activities, family size, experiencing major events in the past six months, frequency of contact with fathers, video and text message communication methods, strict discipline, democratic communication, and permissive education methods (all *P* < 0.05). However, among male LBMSSs, there were statistically significant differences in anxiety subgroups regarding experiencing major events in the past six months and frequency of contact with mothers (all *P* < 0.05), as shown in **Table 1**.

**Table 1:** Basic information on girls and boys in social anxiety groups.

Variables	Subgroup	Girls				Boys			
		No-SA	SA	*t*, *χ* ^2^	*P*	No-SA	SA	*t*, *χ* ^2^	*P*
Age		14.04 ± 0.96	14.01 ± 1.05	0.311	0.756	14.02 ± 1.02	14.09 ± 0.94	0.468	0.446
Class	Seven	50	41	1.330	0.514	84	44	1.308	0.520
	Eight	59	66			85	47		
	Nine	69	64			102	69		
Extracurricular Activities	No	15	37	12.004	0.001	23	17	0.546	0.460
	Yes	163	134			248	143		
Family Size		4.93 ± 1.37	4.62 ± 1.41	2.102	0.036	4.76 ± 1.37	4.87 ± 1.44	-0.753	0.452
Siblings		2.03 ± 0.55	1.98 ± 0.8	0.776	0.438	1.91 ± 0.81	1.91 ± 0.64	-0.014	0.989
Suffered major incidents in the last six months	No	163	142	5.840	0.016	242	126	8.970	0.003
	Yes	15	29			29	34		
Migrant Workers	Parents	136	127	0.258	0.879	216	124	2.051	0.359
	Father	36	37			46	26		
	Mother	6	7			9	10		
Father	Non	6	7	0.127	0.722	9	10	2.048	0.152
	Yes	172	164			262	150		
Working Place	Inside province	68	66	0.018	0.894	112	60	0.296	0.586
	Outside province	104	98			150	90		
Frequency of Returning Home	Never	1	7	8.571	0.073	1	1	0.974	0.914
	1/year	99	105			149	90		
	1/half year	36	29			62	32		
	1/season	17	13			26	16		
	1/month	19	10			24	11		
Frequency of Contacting	Never	7	19	17.670	0.007	10	8	10.620	0.101
	1/year	0	1			2	0		
	1/half year	4	1			5	0		
	1/season	1	5			2	5		
	1/month	12	20			28	23		
	1/week	130	109			192	104		
	1/day	18	9			23	10		
Mother	Non	39	38	0.005	0.944	53	26	0.735	0.391
	Yes	139	133			218	134		
Working Place	Inside province	59	49	0.718	0.397	99	59	<0.001	0.996
	Outside province	83	85			126	75		
Frequency of Returning Home	Never	9	7	3.127	0.537	17	8	4.348	0.361
	1/year	72	75			112	78		
	1/half year	30	33			46	29		
	1/season	14	9			25	10		
	1/month	17	10			25	9		
Frequency of Contacting	Never	5	12	11.333	0.079	7	6	14.782	0.022
	1/year	0	2			2	3		
	1/half year	3	0			4	1		
	1/season	0	2			0	5		
	1/month	6	8			23	22		
	1/week	116	99			168	88		
	1/day	12	11			21	9		
Contacting Method									
Telephone	Non	42	39	0.030	0.862	56	31	0.104	0.747
	Yes	136	132			215	129		
Video	Non	26	41	4.936	0.026	43	36	2.957	0.086
	Yes	152	130			228	124		
Text massage	Non	45	29	3.615	0.057	82	37	2.561	0.110
	Yes	133	142			189	123		
Guardian	Parents	16	13	0.512	0.774	26	14	1.382	0.501
	Grandparents	154	148			225	129		
	Other Relatives	8	10			20	17		
Education Level	Never	14	10	1.687	0.194	26	20	0.142	0.706
	Primary	97	113			137	78		
	Junior	56	39			89	49		
	Senior and above	11	8			19	13		
Parenting style									
Strict Control	Non	133	107	5.991	0.014	192	105	1.281	0.258
	Yes	45	64			79	55		
democratic communication	Non	21	36	5.467	0.019	38	31	2.144	0.143
	Yes	157	135			233	129		
Neglectful Parenting	Non	156	135	4.756	0.029	236	134	0.921	0.337
	Yes	22	36			35	26		

SA, Social Anxiety.

In girls, Spearman’s test shown the SA is related to family size (*r*_s_=-0.128, *P* = 0.017), father out (*r*_s_=-0.173, *P* = 0.001), mother out (*r*_s_=-0.141, *P* = 0.008), father returning home frequency (*r*_s_=-0.131, *P* = 0.016), life satisfaction (*r*_s_=-0.128, *P* = 0.017), extracurricular activities (*r*_s_=-0.224, *P* < 0.001), father contacting frequency (*r*_s_=-0.187, *P* < 0.001), Video communication (*r*_s_=-0.119, *P* = 0.026), Neglecting parenting (*r*_s_=-0.117, *P* = 0.026), Suffering major events (*r*_s_=-0.128, *P* = 0.016), democratic communication (*r*_s_=-0.125, *P* = 0.019) and Strict Control (*r*_s_=-0.131, *P* = 0.014). For boys, SA is related to mother contacting frequency (*r*_s_=-0.134, *P* = 0.011), suffering major events (rs=-0.144, P = 0.003), and democratic communication (*r*_s_=-0.143, *P* = 0.007).

**Table 2** shows that female participation in extracurricular activities (*OR =* 0.301, *P =* 0.005), frequency of father contact (*OR =* 0.817, *P =* 0.035), and video communication (*OR =* 0.423, *P =* 0.021) are protective factors against SA. At the same time, neglect (*OR =* 2.170, *P =* 0.032) is a risk factor for SA, with *R*^2^ = 0.145. For males experiencing major events (*OR =* 2.662, *P =* 0.001) is a risk factor for SA, whereas democratic communication with parents is a protective factor (*OR =* 0.538, *P =* 0.037), with *R*^2^ = 0.074.

**Table 2:** Risk factor analysis of social anxiety in girls and boys by the binary logistic regression method.

Gender	Variable	*OR* (95% CI)	*P*	SE	Wald *χ*^2^
Girls	Extracurricular Activities	0.301 (0.131, 0.694)	0.005	0.426	7.935
	Frequency of Contacting with Father	0.817 (0.677, 0.986)	0.035	0.096	4.424
	Video	0.423 (0.204, 0.879)	0.021	0.373	5.314
	Neglectful Parenting	2.170 (1.070, 4.398)	0.032	0.360	4.617
Boys	Suffered major incidents	2.662 (1.457, 4.863)	0.001	0.307	10.142
	Democratic Communication	0.538 (0.300, 0.964)	0.037	0.298	4.338

## Discussion

The findings of this study suggest the presence of substantial gender disparities in the risk factors associated with social anxiety levels among LBMSSs. Specifically, among females, active engagement in extracurricular activities, frequent contact with fathers, and communication via video may be positively related to the risk of SA. Conversely, neglecting parenting has been found to be associated with the increased probability of SA occurrence markedly. Among males, the occurrence of crucial adverse life events (e.g., the death of a loved one) within the past six months is associated with an increased propensity to experience SA. Conversely, cultivating democratic communication with parents may be negatively associated with the risk of SA.

### Girls: extracurricular activities

Girls are more prone to the anxiety of her friends than boys (11) due to the empathy, but seldom accept their anxious status (12). Furthermore, girls have been found to be more sensitive to unfavorable evaluations, such as rejection and appearance ridicule, in social activities. This can more easily lead to social withdrawal and social anxiety (13). However, Extracurricular activities facilitate the cultivation of shared interests and hobbies among LBC and their peers, thereby enhancing friendship and interpersonal relationships, and promoting the development of their interpersonal communication skills. Research has demonstrated that different types of extracurricular activities can exert interactive effects on adolescents’ social-emotional competence (14). Specifically, incorporating physical exercise has been shown to enhance self-efficacy and psychological resilience in this demographic (15). Furthermore, active engagement in social media has been demonstrated to play a pivotal role in the mitigation of depression and anxiety in adolescents (16, 17). This study further finds that participation in extracurricular activities may also be related to the reduced risk of SA among female LBC. Consequently, the government and educational institutions should prioritize the development and provision of suitable extracurricular activities for LBC. Furthermore, it is essential to ensure that these activities are free from any form of differential treatment or discrimination during implementation (18).

### Girls: contacting father frequently

Parents can encourage children to engage in daily skill development, collaborative exploratory activities, and the resolution of interpersonal conflicts, to the promotion of cognitive development and the regulation of emotional responses (19). For instance, empirical evidence suggests a positive correlation between the level of interaction between fathers and their children and young children’s emotional regulation ability (20). The intimacy of father-child relationships evolves, with a marked increase observed in late adolescence. Still, a decrease in intimacy may lead to heightened anxiety levels in girls in early adolescence and an elevated risk of depression in mid-adolescence (21). Furthermore, the absence of paternal care during early development may induce impairment of neurodevelopment and socioemotional development in children (22), resulting in more likely to develop depression symptoms in girls than in boys (23). In addition, the care from a father has a significant impact on the establishment of self-esteem in his daughter during junior high school (24). The findings outlined above may lend support to our results that the maintenance of frequent contact between fathers and daughters may contribute to a reduction in the risk of adolescent girls developing symptoms of social anxiety (SA). This suggests a potential importance of paternal engagement in the mental health development of daughters.

### Girls: video communication method

Research has demonstrated that video communication can enhance intimate relationships between parents and children through the “body extension” effect and emotional reinforcement (25). In contrast, parent-child communication facilitated by videoconferencing can enhance children’s physical and mental wellbeing (26). Video communication has been shown to provide richer emotional cues and more direct emotional support, especially as the efficacy of language communication will influence children’s mental health (27). Furthermore, girls have been shown to be more sensitive to emotional changes (13). Consequently, the utilization of video communication may enhance safety and wellbeing (28), resulting in mitigating the risk of social anxiety among girls. Interestingly, video communication has the potential to enhance the sensitivity of parents with regard to identifying the mental health problem (29). Consequently, promoting visual media as a primary means of daily communication between migrant parents and their children has the potential to enhance the mental wellbeing of left-behind adolescents, particularly female adolescents.

### Girls: neglecting parenting

The neglected issue of LBC in China encompasses a wide range of challenges, including but not limited to the emotional, physical, educational, safety, medical, and social support needs of these children (1). Families frequently prioritize educational investment for boys, while girls may be implicitly expected to assume household chores or to prepare for marriage as early as possible (30). This implicit neglect limits their opportunities to develop social skills, thereby placing them at a disadvantage in collective settings such as schools. This, in turn, further undermines their social confidence (31). In the absence of their parents for extended periods, these children frequently encounter difficulties in establishing secure attachment patterns (32). Consequently, when navigating peer social environments, they exhibit a dualistic tendency: a fervent desire for interpersonal connections, coupled with a concurrent reluctance to disclose their emotional vulnerabilities. This contradictory mindset is prone to manifesting as social avoidance and anxiety behaviors (33).

### Boys: suffered major incidents

Under the influence of traditional gender socialization, male LBC may be expected to embody qualities of “strength” and “resilience,” and their emotional expression and propensity to seek assistance may be repressed (34). This phenomenon contributes to the internalization of anxiety and avoidance, rather than the externalization or confiding that is typically associated with processing trauma (35). In addition, significant events can directly result in traumatic reactions and potentially subvert individuals’ fundamental assumptions regarding the safety and controllability of the world (36). Major adverse events do not act in isolation but rather function as crucial catalysts, interacting with existing risk factors in the left-behind environment (e.g., poor guardianship quality and weak social support networks) to collectively propel individuals toward the pathological threshold of social anxiety (37). This finding underscores the necessity for conducting trauma screening and establishing targeted psychological crisis intervention mechanisms for this high-risk group. Intervention measures must integrate trauma-focused cognitive behavioral therapy and enhance social support systems to block this risk pathway.

### Boys: democratic communication

Democratic communication, characterized by mutual respect, emotional warmth, open discussion, and joint decision-making, has been identified as a potential solution to address the challenges faced by left-behind adolescents in a parent-child separation context (38). This approach aims to foster a stable and responsive emotional connection, thereby offering a partial compensation for the absence of physical companionship (39). When parents affirm their children’s viewpoints and encourage them to express themselves in communication, democratic communication, as a continuous form of social interaction, provides adolescents with templates for observing and learning adaptive social skills, emotion regulation strategies, and constructive conflict-resolution methods (40), resulting in the improvement of individuals’ self-efficacy and sense of value (41). Repeated positive and high-quality parent-child interactions lead to the internalization of a more secure internal working model, thereby reducing cognitive biases that perceive social interactions as threatening (42). Consequently, democratic communication is not merely a communication skill; it is also a pivotal relational resource.

### Limitations

The limitations are as follows. Firstly, the cross-sectional design employed precludes causal inference. Secondly, although stratified sampling was used, the generalizability of the results may still be limited by geographical constraints. Thirdly, the data is based on self-administered questionnaires, which may be subject to recall bias and social desirability bias. Moreover, the study did not evaluate other potential influential factors, including the school environment, peer relationships (43), and community support. In the future, multicenter prospective studies that integrate qualitative interviews with objective physiological indicators are scheduled in plan.

## Conclusion

This study analyzed the gender-specific risk factors of SA in LBMSSs, revealing possible differences in the formation mechanism of SA between boys and girls. These findings suggest that future mental health interventions for LBC should adopt gender-sensitive strategies, implementing targeted prevention and support measures through multi-party collaboration among families, schools, and communities to alleviate their social anxiety and promote mental health development.

## Data Availability

The raw data supporting the conclusions of this article will be made available by the authors, without undue reservation.
