# Supplementary material for: Analysis of the gender-specific risk factors of social anxiety among left-behind middle school students in Deyang
Source: Front Psychiatry. 2026 Mar 25;17:1780497. doi: 10.3389/fpsyt.2026.1780497 (PMC13058603; doi:10.3389/fpsyt.2026.1780497)
Supplement: Supplementary file 1 [file Supplementaryfile1.docx]

**Supplementary 1. The sample size estimation method**

This study primarily employs a survey-based sampling approach among left-behind middle school students in grades 7-9 from various towns and townships in Zhongjiang. It intends to use stratified random sampling, stratifying by the number of students in each city in Zhongjiang County, with three levels: “<500”, “500-1000”, and “>1000”. Using the formula for estimating the sample size required to calculate the population rate (as shown below) and adopting incomplete statistical data from the Education Bureau of Zhongjiang, the number of children in grades 7-9 in Zhongjiang is 31,639, of whom 3,862 are left behind middle school students. The allowable error is set at 2%, and the confidence level is 95%. According to the formula, the number of left-behind children in grades 7-9 in Zhongjiang County to be surveyed is 856. Considering a 10% dropout rate, the estimated number of left-behind children in grades 7-9 in Zhongjiang County to be sampled for the survey is 951.

(1) Formula for calculating total sample size:

***N****_i_* and *p*_i_ denote the number and proportion of left-behind middle school students in the *i*_th_ school, respectively; ***V*** denotes the estimated variance of the population rate; and ***N*** represents the total number of middle school students in Zhongjiang.

The sample size of each stratum is shown in **Table S1**.

| **Table S1. The sample size of each stratum.** | | | | | | | | |
| --- | --- | --- | --- | --- | --- | --- | --- | --- |
| **Stratum** | ***N*** | ***n_i_*** | ***p_i_*** | ***W_i_*** |  |  |  | ***N_i_*** |
| <500 | 8939 | 1587 | 0.18 | 0.411 | 0.157 | 0.060 | 606.428 | 461 |
| 500-1000 | 9588 | 1327 | 0.14 | 0.344 | 0.119 | 0.041 | 458.242 | 349 |
| >1000 | 13112 | 948 | 0.07 | 0.245 | 0.064 | 0.016 | 60.267 | 46 |
|  | 31639 | 3862 |  | 1 | 0.339 | 0.117 | 1124.936 | 856 |

(2) Formula for calculating the sample size of each stratum:

The detailed estimated number of students investigated is shown in **Table** **S2**.

| **Table S2**. The detailed sample size of each investigated school. | | | | | |
| --- | --- | --- | --- | --- | --- |
| Schools | Left behind students | Total Students | Stratification | Estimated | Plus 10% dropout |
| CS | 36 | 236 | <500 | 18 | 20 |
| JGSY | 22 | 366 | <500 | 10 | 12 |
| JNWG | 76 | 347 | <500 | 38 | 42 |
| LHCJ | 62 | 349 | <500 | 30 | 34 |
| LHQX | 48 | 156 | <500 | 23 | 26 |
| LT | 50 | 310 | <500 | 25 | 28 |
| PX | 129 | 354 | <500 | 64 | 71 |
| QSCJ | 35 | 189 | <500 | 17 | 19 |
| SL | 56 | 214 | <500 | 28 | 31 |
| TAI | 87 | 305 | <500 | 43 | 48 |
| TSX | 54 | 205 | <500 | 27 | 30 |
| WF | 89 | 429 | <500 | 44 | 49 |
| YF | 92 | 284 | <500 | 46 | 51 |
| YL | 76 | 460 | <500 | 38 | 42 |
| ZJSY | 17 | 429 | <500 | 9 | 10 |
| GF | 212 | 857 | 500-1000 | 149 | 165 |
| NH | 85 | 567 | 500-1000 | 60 | 66 |
| YTZ | 98 | 955 | 500-1000 | 70 | 76 |
| YXZ | 102 | 825 | 500-1000 | 72 | 80 |
| XL | 133 | 1942 | >1000 | 46 | 51 |
| **Total** | **1559** | **9779** |  | 856 | **951** |


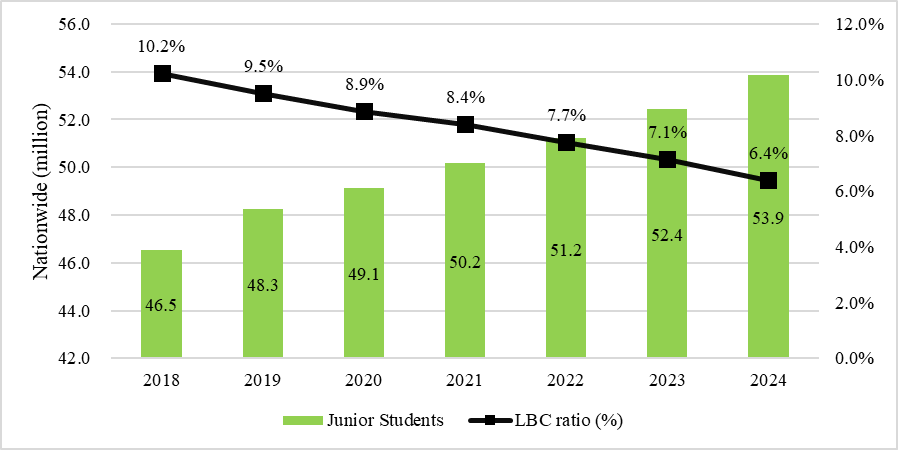


**Figure S1A**. Changes in the proportion of left-behind middle school students nationwide from 2018 to 2023.


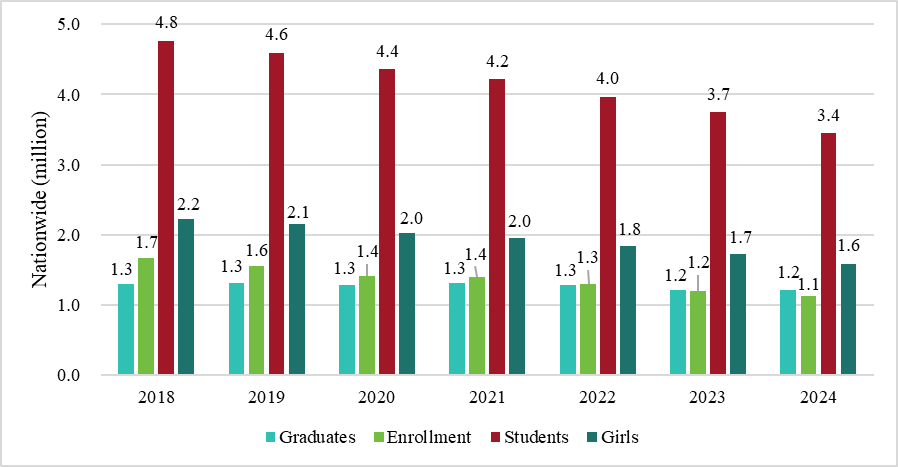


**Figure S1B**. The annual number of left-behind middle school students graduating, the proposed number of recruits, the number of students currently enrolled, and the number of females presently enrolled from 2018 to 2023.
