# Supplementary material for: Analysis of the gender-specific risk factors of social anxiety among left-behind middle school students in Deyang
Source: Front Psychiatry. 2026 Mar 25;17:1780497. doi: 10.3389/fpsyt.2026.1780497 (PMC13058603; doi:10.3389/fpsyt.2026.1780497)
Supplement: Supplementary file 2 [file Supplementaryfile2.docx]

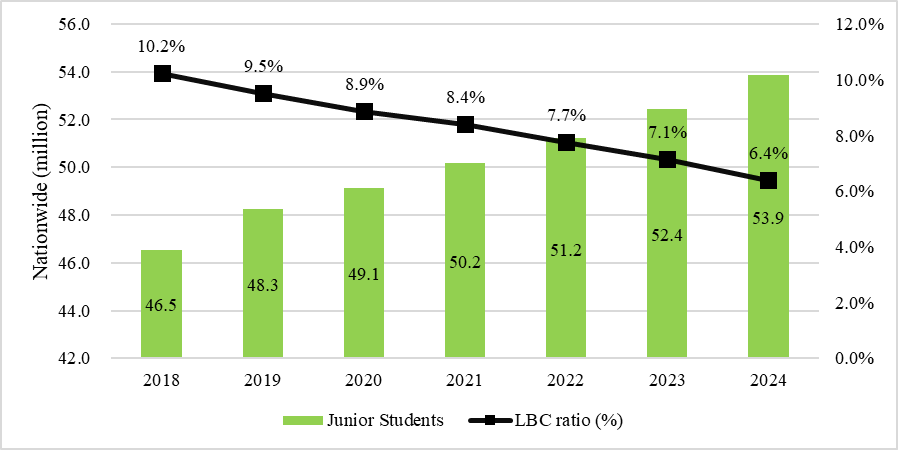


**Figure S1A**. Changes in the proportion of left-behind middle school students nationwide from 2018 to 2023.


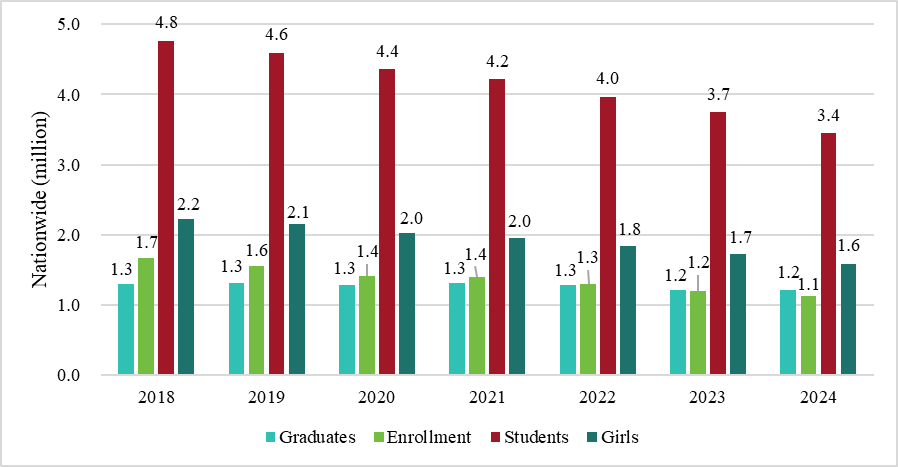


**Figure S1B**. The annual number of left-behind middle school students graduating, the proposed number of recruits, the number of students currently enrolled, and the number of females presently enrolled from 2018 to 2023.
